# A single dose of replication-competent VSV-vectored vaccine expressing SARS-CoV-2 S1 protects against virus replication in a hamster model of severe COVID-19

**DOI:** 10.1038/s41541-021-00352-1

**Published:** 2021-07-22

**Authors:** Delphine C. Malherbe, Drishya Kurup, Christoph Wirblich, Adam J. Ronk, Chad Mire, Natalia Kuzmina, Noor Shaik, Sivakumar Periasamy, Matthew A. Hyde, Julie M. Williams, Pei-Yong Shi, Matthias J. Schnell, Alexander Bukreyev

**Affiliations:** 1 Department of Pathology, University of Texas Medical Branch, Galveston, TX USA; 2 Galveston National Laboratory, Galveston, TX USA; 3 Department of Microbiology and Immunology, Thomas Jefferson University, Philadelphia, PA USA; 4 Department of Microbiology & Immunology, University of Texas Medical Branch, Galveston, TX USA; 5 Department of Biochemistry and Molecular Biology, University of Texas Medical Branch, Galveston, TX USA; 6 Institute for Human Infections and Immunity, University of Texas Medical Branch, Galveston, TX USA; 7 Jefferson Vaccine Center, Thomas Jefferson University, Philadelphia, PA USA

**Keywords:** SARS-CoV-2, Live attenuated vaccines

## Abstract

The development of effective countermeasures against severe acute respiratory syndrome coronavirus 2 (SARS-CoV-2), the agent responsible for the COVID-19 pandemic, is a priority. We designed and produced ConVac, a replication-competent vesicular stomatitis virus (VSV) vaccine vector that expresses the S1 subunit of SARS-CoV-2 spike protein. We used golden Syrian hamsters as animal models of severe COVID-19 to test the efficacy of the ConVac vaccine. A single vaccine dose elicited high levels of SARS-CoV-2 specific binding and neutralizing antibodies; following intranasal challenge with SARS-CoV-2, animals were protected from weight loss and viral replication in the lungs. No enhanced pathology was observed in vaccinated animals upon challenge, but some inflammation was still detected. The data indicate rapid control of SARS-CoV-2 replication by the S1-based VSV-vectored SARS-CoV-2 ConVac vaccine.

The current pandemic of coronavirus-induced disease 2019 (COVID-19) caused by severe acute respiratory syndrome coronavirus 2 (SARS-CoV-2) has resulted in over 146 million confirmed cases of human infections worldwide, including over three million confirmed deaths (as of April 25, 2021)^1^. A few vaccines have been approved and many more are being developed, including those based on mRNA, viral vectors, inactivated virus, and protein subunits^2,3^. The main antigen of these vaccines is the full-length spike (S) protein of SARS-CoV-2, since the virus uses its S protein to mediate cell entry.

Nonsegmented negative-strand RNA viruses including vesicular stomatitis virus (VSV), which replicates systemically, represent highly potent vaccine platforms^4^. These vaccine platforms are capable of stably expressing foreign open-reading frames flanked with transcriptional gene-start and gene-end signals specific for the viral vector’s polymerase. A number of vaccine candidates for Middle East respiratory syndrome coronavirus (MERS-CoV), SARS-CoV, SARS-CoV-2, and porcine coronavirus utilized VSV and rabies virus as vector backbones to express the spike protein as immunogen^5–9^. We previously developed a MERS vaccine candidate in which the S1 domain of the MERS spike protein was fused to the C-terminal part of the rabies virus glycoprotein to allow incorporation and display on the surface of virions^9^. We reasoned that removal of the S2 domain should steer the immune response toward the receptor-binding domain that is located in the S1 domain and is the target of neutralizing antibodies. To expedite COVID-19 vaccine development, we took this proven strategy to generate a SARS-CoV-2 vaccine candidate in which the S2 domain was replaced with the C-terminal part of the VSV glycoprotein as a membrane anchor and expressed in a replication-competent VSV vector.

We assessed the immunogenicity and efficacy of our vaccine in the recently established golden Syrian hamster model of COVID-19^10–12^. Golden Syrian hamsters are susceptible to intranasal infection with wild-type SARS-CoV-2 without the need for virus adaptation, and develop severe clinical manifestations similar to those observed in human COVID-19 patients with a severe disease^10–12^.

## Results

### Design and development of the VSV-SARS-CoV-2 vaccine ConVac

To design our ConVac vaccine construct, we decided to use only the S1 domain of SARS-CoV-2 spike S for several reasons. First, the S1 region contains the necessary neutralizing epitopes with the receptor-binding domain being most important^13^. Second, both prefusion and postfusion S2 structures present potential drawbacks as vaccine constructs, and vaccines expressing the full-length S-spike protein may generate both prefusion and postfusion spikes as observed in in vitro mammalian cell culture production systems^14^. Because of its high level of glycosylation it has been proposed that the S2 postfusion configuration may act as an immunological decoy by eliciting immunodominant nonneutralizing responses while stabilization of prefusion S2 may affect bonds between protomers thus affecting the overall trimer stability^14^. The third reason for the use of the S1-only approach is that the S2 domain is more conserved between different coronaviruses^15,16^, and we hypothesized that in previously coronavirus-infected humans the immune response might predominantly target S2 rather than S1. Last, previous research with a rabies virus-vectored vaccine indicated that the expression of the full-length coronavirus S protein interfered with the transport of the rabies virus G protein and reduced vaccine viral titers dramatically^9^. Moreover, VSV G-deleted viruses grow to a lower titer of about 10^7^ PFU, whereas VSV G-containing viruses can reach titers up to 1–5 × 10^8^, making vaccine production more efficient^17,18^. These reasons led us to develop a VSV-based vaccine construct containing only the membrane-anchored S1 domain by substituting the S2 domain of the SARS-CoV-2 spike protein with the C-terminal region of the VSV glycoprotein.

**Fig. 1:**
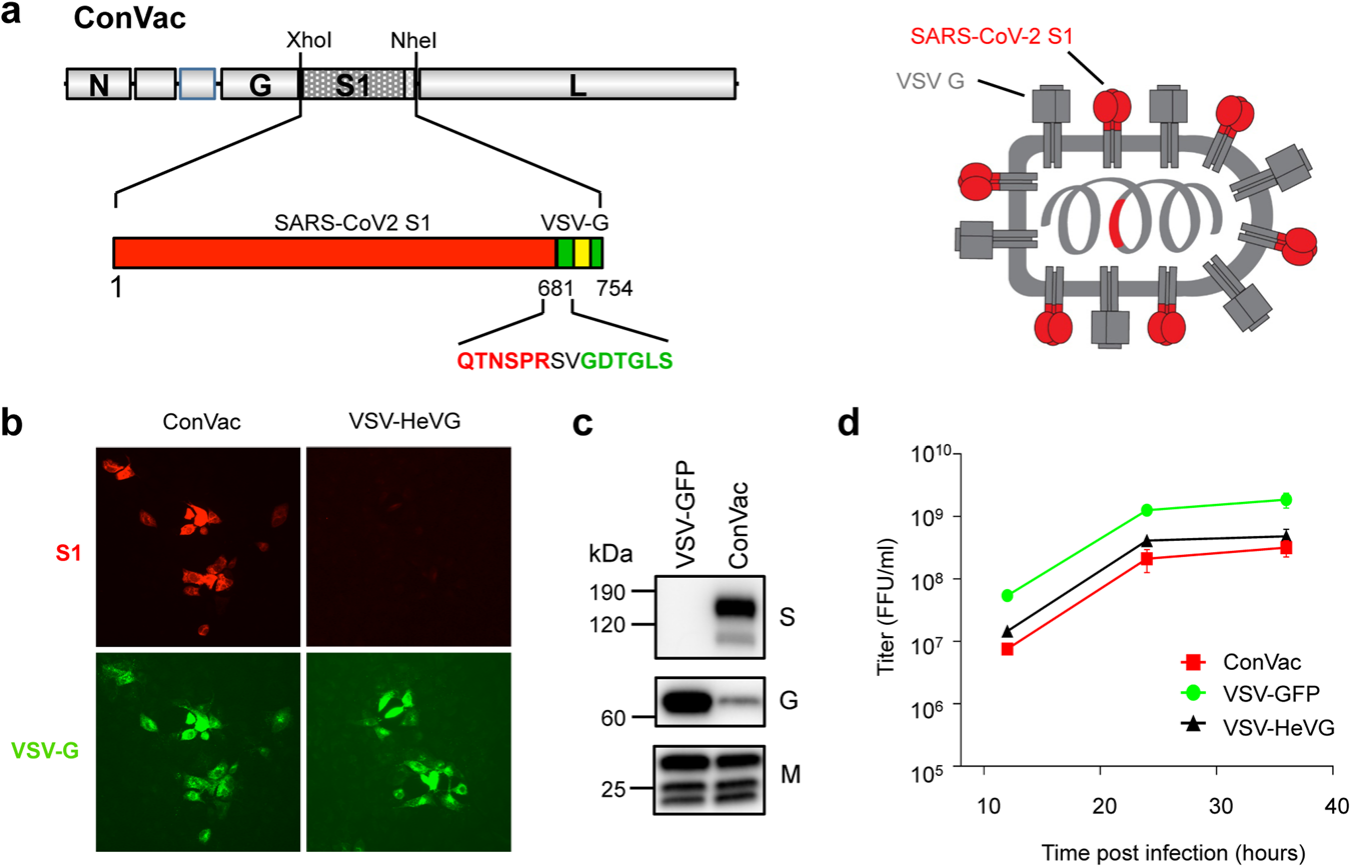
Generation and characterization of ConVac. **a** Left: genome structure of the VSV vector expressing the membrane-anchored SARS-CoV2 S1 domain. The S1 domain of SARS-CoV2 spike protein (aa 1–681) was joined to the C-terminal 70 amino acids of the VSV glycoprotein. The fusion construct was inserted between the glycoprotein and polymerase genes of VSV. The S1 domain is shown in red and the VSV G tail in green. The transmembrane domain of the VSV glycoprotein is indicated by a yellow box. The amino acids at the junction between S1 and VSV glycoprotein are highlighted. Right: schematic representation of the vaccine construct that shows the two transmembrane proteins anchored in the membrane. **b** Immunofluorescence staining of Vero E6 cells infected with ConVac and a control virus expressing Hendra virus G (VSV-HeVG). The cells were fixed and permeabilized 10 h after infection and stained with fluorescently labeled monoclonal antibody CR3022 against the S1 domain (shown in red) and two monoclonal antibodies against the VSV glycoprotein (shown in green). **c** Western blot analysis of BSR cells infected with Convac and a control VSV virus expressing GFP. Protein lysates were resolved on 4–20% polyacrylamide-gradient gels and transferred to nitrocellulose membranes. The membranes were probed with polyclonal antiserum against the S1 domain (upper panel), monoclonal antibodies against the VSV glycoprotein (middle panel), and a monoclonal antibody against the VSV matrix protein (lower panel). VSV glycoprotein expression was significantly reduced in cells infected with ConVac whereas matrix protein expression was only modestly affected. **d** Viral growth curve on Vero cells. The cells were infected at an MOI of 0.05 PFU with ConVac, or VSV-expressing GFP or another control virus expressing Hendra virus glycoprotein. Supernatants were collected 12, 24, and 36 h post infection and titrated on Vero E6 cells. Data represent mean ± SD.

Thus, following the strategy we previously employed to generate a membrane-anchored S1 domain of MERS^9^, and we replaced the S2 domain of SARS-CoV-2 (aa 682–1284) with the C-terminal 70 amino acids of VSV G (Fig. 1a). This portion contains the complete cytoplasmic tail of the VSV glycoprotein, as well as the transmembrane domain and a short membrane-proximal portion of the ectodomain of VSV-G. The VSV-G tail serves as a membrane anchor for S1 and allows incorporation into VSV particles. We recovered the virus as described previously^19^ and performed immunofluorescence staining with antibodies directed against S1 or VSV glycoprotein (Fig. 1b). Vero cells infected with ConVac expressed both S1 and VSV glycoprotein. No S1 signal was detected in cells infected with a control virus expressing Hendra virus G. We also performed western blotting to assess protein expression (Fig. 1c, S1). Using polyclonal antiserum directed against the S1 domain we detected a protein of 160 kDa and a shorter band of approximately 100 kDa. The smaller band presumably represents unglycosylated or partially glycosylated protein, whereas the larger band represents the fully glycosylated mature protein. VSV glycoprotein expression was noticeably reduced compared with the control virus-expressing GFP. To assess the impact on viral replication, we evaluated growth kinetics in Vero cells (Fig. 1d). ConVac grew to titers 2–4 × 10^8^ FFU/ml, which are about five-fold lower than the control virus expressing-GFP and modestly lower compared with a recombinant VSV-expressing Hendra virus G. Similar titers were obtained in human lung and baby hamster kidney cells (data not shown).

### Development of the hamster model of COVID-19

**Fig. 2:**
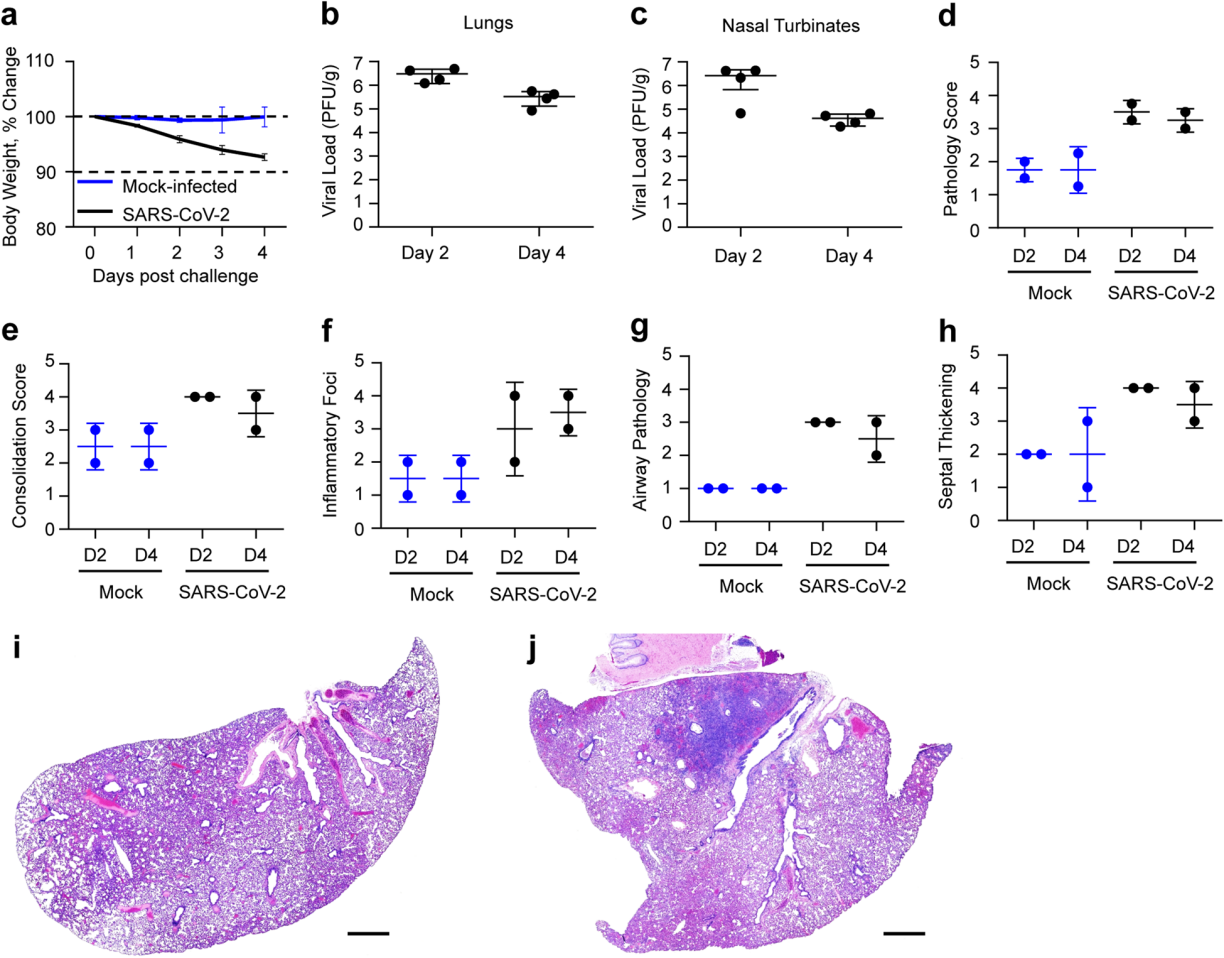
Pathogenicity and replication of SARS-CoV-2 in golden Syrian hamsters. Hamsters were challenged intranasally with 10^5^ PFU SARS-CoV-2 at day zero and monitored for four days. **a** % change in body weight. Data represent mean ± SEM. SARS-CoV-2 group is shown in black, and mock infected group in blue. Viral loads were determined in lung **b** and nasal turbinate **c** tissues two and four days post challenge. Right lungs and nasal turbinates from each animal were homogenized in media and titrated on Vero E6 cells. No virus was detected in the mock-infected animals (data not shown). *N* = 4 for mock-control group (two hamsters euthanized at each timepoint) and *N* = 8 for VSV vaccine group (four hamsters euthanized at each timepoint). Comparative pathology scores for lungs from SARS-CoV-2-infected and mock-infected hamsters (two animals in each group) were determined 2 days (D2) and 4 days (D4) post challenge (**d**–**h**). Scores for overall lung pathology (**d**), and individual criteria including consolidation or extent of inflammation (**e**), type of inflammatory foci (**f**), airway pathology (**g**) and septal thickening (**h**) are displayed. The pathology scores (mean) were calculated based on the criteria described in Supplementary Table 1. Data represent mean ± SD, *N* = 2 for each group at each timepoint. **i** Day four mock-infected lung displaying minimal pathologic changes in airways. Note a septal thickening and alveolar wall damage (likely due to CO_2_ euthanasia). **j** Day four SARS-CoV-2-infected lung displaying widespread inflammatory change, septal thickening, and airway infiltration. Scale bars are 1 mm.

To determine if golden Syrian hamsters are a suitable animal model to study the protective efficacy of the vaccine, eight female golden Syrian hamsters were infected intranasally with 10^5^ PFU of SARS-CoV-2 strain USA-WA1/2020^20^ at day zero in a volume of 100 µl, and four female hamsters were mock-infected with 100 µl of phosphate-buffered saline. Animals were monitored daily for weight loss (Fig. 2a). Viral load determination in the lungs (Fig. 2b) and nasal turbinates (Fig. 2c) was performed two and four days post challenge. At day four, SARS-CoV-2-infected hamsters had lost an average of 7.3% of their initial body weight, while mock-infected animals did not display any weight loss (Fig. 2a). Viral loads were both higher in the lungs (mean viral load of 3.0 × 10^6^ PFU/g) and in the nasal turbinates (2.7 × 10^6^ PFU/g) at day two compared with day four (3.4 × 10^5^ PFU/g of lung and 4.1 × 10^4^ PFU/g nasal turbinate) (Fig. 2b, c). Lung histopathological changes were assessed in two animals of each group at each time point (criteria displayed in Supplementary Table 1^21^). The SARS-CoV-2-infected hamsters displayed lung pathology consistent with typical interstitial pneumonia at both time points (Fig. 2d), while some alveolar changes and septal thickening (Fig. 2i) were also observed in the mock-infected animals that were a consequence of the CO_2_ euthanasia method used in this pilot study. In SARS-CoV-2-infected hamsters, widespread inflammatory changes consisting of small-to-large inflammatory foci were observed (Fig. 2e, f). In addition, infiltration of airways with inflammatory cells (Fig. 2g, j) and a moderate level of inter-alveolar septal thickening were noted in SARS-CoV-2-infected animals (Fig. 2h, j). Thus, lung pathology observed in our pilot study indicates that the golden Syrian hamster is a suitable animal model reproducing the typical interstitial pneumonia caused by SARS-CoV-2 in humans. In addition, our findings are in agreement with others as while this paper was in preparation, other teams reported that SARS-CoV-2 infection of hamsters causes a severe lung disease, with viral replication in the upper and lower respiratory tract^10–12^.

### ConVac vaccine induces a robust antibody response

**Fig. 3:**
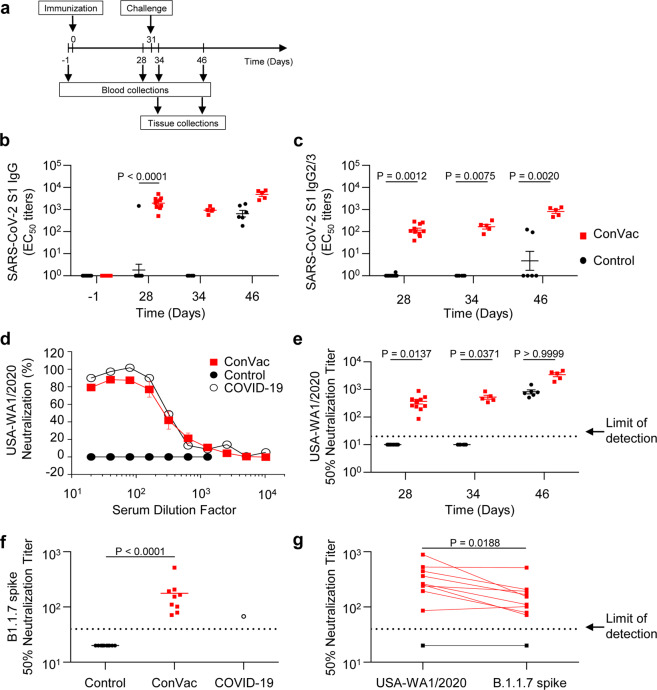
ConVac elicits SARS-CoV-2 S1-specific binding and neutralizing antibodies. **a** Study schedule. Sera collected during the immunogenicity phase (days minus one and day 28) and the challenge phase (day 34 is three days post challenge and day 46 is 15 days post challenge) were assessed for their ability to bind to SARS-CoV-2 spike protein S1 and to neutralize SARS-CoV-2. **b**, **c** Antibody responses determined by ELISA: total IgG (**b**) and IgG2/3 (**c**) displayed with a line at mean ± SEM and *P* values determined by Kruskal–Wallis test, followed by Dunn’s multiple comparison. **d** Neutralization of the USA-WA1/2020 virus at day 28 displayed as mean ± SEM. A plasma sample from a COVID-19 convalescent human subject was included as positive control. **e** Longitudinal 50% neutralization titers against the USA-WA1/2020 virus with line at mean ± SEM with P values determined by Kruskal–Wallis test, followed by Dunn’s multiple comparison. **f** Neutralization of the UK (B.1.1.7) virus by day-28 serum samples, with line at mean. *P* value determined by Mann–Whitney test. **g** Comparison of neutralization of the USA-WA1/2020 virus and the UK (B.1.1.7) virus shown for individual animals. *P* value for ConVac determined by Mann–Whitney test.

The immunogenicity and efficacy of the ConVac vaccine was evaluated in the golden Syrian hamster animal model. Hamsters at 12 animals per group were vaccinated once intramuscularly on day zero (Fig. 3a) with 2 × 10^7^ FFU/animal of ConVac, while the control group remained naive. On day 10 post vaccination, one vaccinated hamster was euthanized due to hind-leg paralysis, and on day 11, another hamster was found dead after being lethargic, scruffy, hunched and with heavy breathing the day before. All other animals demonstrated no disease symptoms. At day 31, vaccinated and control hamsters were challenged intranasally with a dose of 10^5^ PFU of the SARS-CoV-2 isolate USA-WA1/2020^20^. Serum samples were assayed longitudinally to determine SARS-CoV-2 S1-specific binding antibody responses. Testing of the binding to purified S1 protein demonstrated induction of IgG EC_50_ titers ranging from 1:505 to 1:5096 with a mean titer of 1:2279 on day 28 after vaccination (Fig. 3b). One control animal demonstrated the presence of SARS-CoV-2 antibodies by ELISA, but was negative for SARS-CoV-2-neutralizing antibodies. All vaccinated animals demonstrated induction of S1-binding Th1-specific IgG2/3 with titers ranging from 1:39 to 1:285 and a mean titer of 1:139 (Fig. 3c), mirroring the total IgG responses albeit at a lower level. Both total IgG and IgG2/3 S1-specific binding antibody titers were significantly higher in the vaccinated animals compared with the control animals, which displayed detectable antibody levels after the challenge (Fig. 3b, c). IgG1 was not analyzed due to the lack of a suitable secondary antibody for hamster IgG1. Neutralization of SARS-CoV-2 USA-WA1/2020 was assessed with the recombinant SARS-CoV-2-expressing Neon Green protein (SARS-CoV-2-mNG)^22^ using the fluorescent signal as a readout for viral replication, thus enabling a fast and quantitative evaluation. Neutralization curves showed similar levels of SARS-CoV-2 antibodies in ConVac-vaccinated animals and a human COVID-19 survivor (Fig. 3d). After the single immunization, the ConVac vaccine elicited 50% SARS-CoV-2-neutralizing titers ranging from 1:85 to 1:886 with the mean titer of 1:369. After the challenge, the levels of neutralizing antibodies were significantly higher in the vaccinated group compared with the control group (Fig. 3e). The SARS-CoV-2 challenge did not elicit an anamnestic antibody response in the ConVac-vaccinated animals, as the binding and neutralizing antibody titers did not increase between the time points before and after the challenge (day 28 vs 34 and day 28 vs 46, *P* > 0.05 for both comparisons for total IgG, IgG2/3, and neutralizing antibodies, Wilcoxon test). In addition, elicitation of neutralizing antibodies against the United Kingdom (UK) (lineage B.1.1.7) SARS-CoV-2 variant was assessed with the recombinant SARS-CoV-2 USA-WA1/2020 virus engineered to express the spike protein of this virus^23^. Neutralizing antibody titers against the B.1.1.7 UK virus were detected in all tested animals (Fig. 3f). In comparison with the USA-WA1/2020 virus, the difference in neutralization titers of the B.1.1.7 UK virus ranged from zero to a six-fold decrease (Fig. 3g). Taken together, our data show that a single-vaccine dose induced a robust SARS-CoV-2-specific antibody response.

### ConVac vaccine significantly decreases SARS-CoV-2 viral load in the lungs

**Fig. 4:**
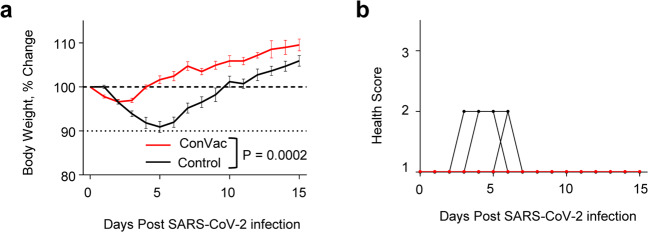
Hamster body weight change and health scores upon SARS-CoV-2 infection. Hamsters were vaccinated at day zero and challenged intranasally with 10^5^ PFU SARS-CoV-2 at day 31. **a** % change in body weight and **b** individual disease score. Data represent mean ± SEM. VSV vaccine is shown in red and control group in black. *N* = 12 for control group (six hamsters euthanized at day three post challenge) and *N* = 10 for ConVac group (five hamsters euthanized at day three post challenge). Body weight *P* value determined by Wilcoxon test.

After the challenge, the hamsters were monitored for 15 days. Animals were checked daily for body weight and clinical signs of disease. In the control group, a significant loss of weight compared with the vaccine group (*P* = 0.0002) was detected, and at the peak of weight loss, which occurred at day five, the average weight loss was 9.1% (Fig. 4a). None of the control or the vaccinated animals reached moribund state, but several control animals had higher disease scores due to a weight loss greater than 10% of their initial body weight (Fig. 4b).

**Fig. 5:**
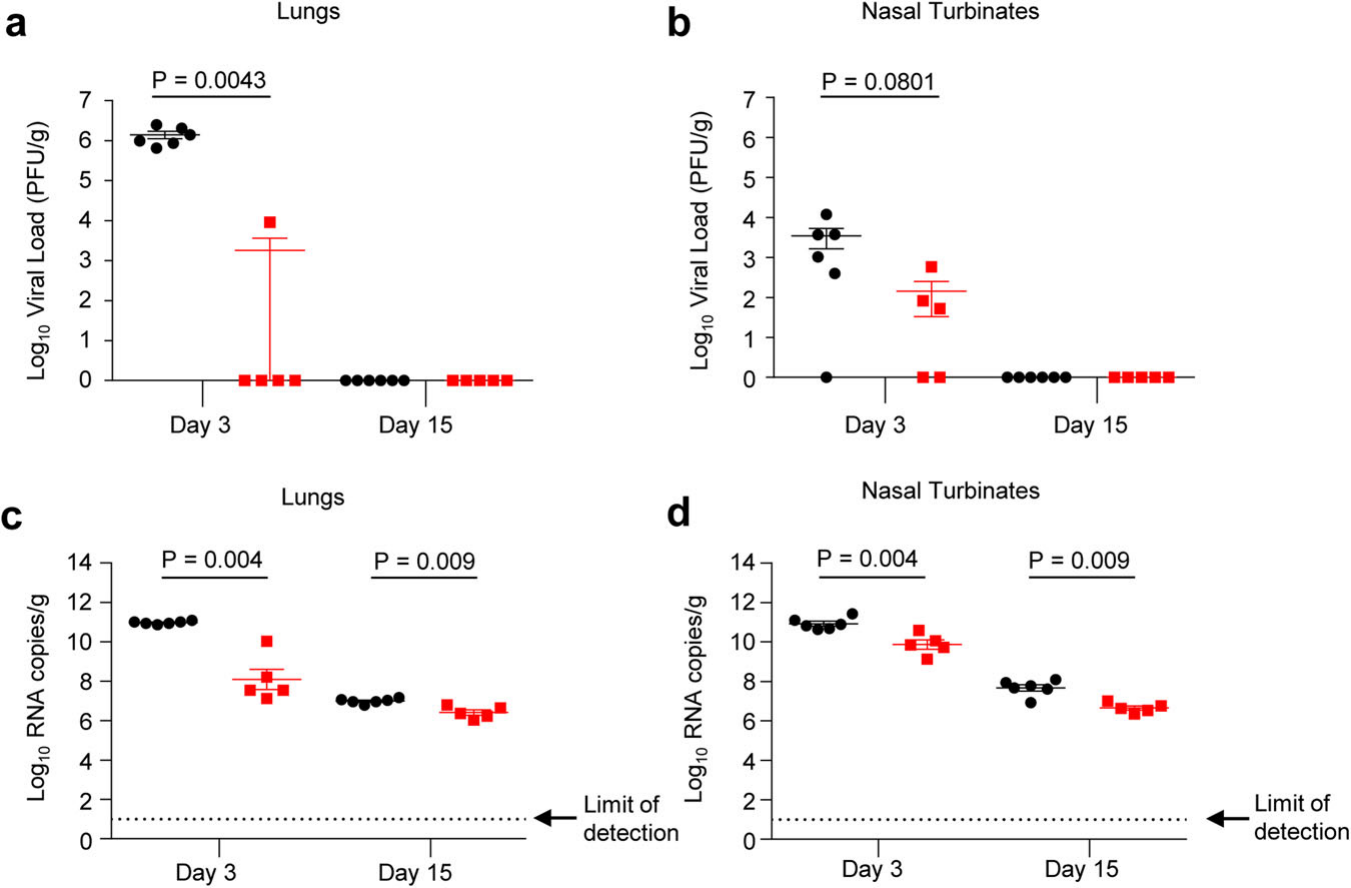
SARS-CoV-2 tissue viral load in hamsters. Hamsters were challenged intranasally with 10^5^ PFU SARS-CoV-2 and half of the animals in each group were euthanized at days three and 15 post challenge. Right lungs (**a**, **c**) and nasal turbinates (**b**, **d**) from each animal were homogenized in media, and viral loads were determined by plaque assays on Vero E6 cells (**a**, **b**) or by qRT-PCR (**c**, **d**). The limit of detection for the plaque assay was 70 PFU per lung and 35 PFU per nasal turbinate. The limit of detection for the qRT-PCR assay is indicated by the dotted line. The ConVac vaccine group is shown in red and the control group in black. For each timepoint, *N* = 6 for control group and *N* = 5 for ConVac vaccine group. Mean values ± SEM. P values determined by Mann–Whitney test.

On days three and fifteen post challenge, half of the hamsters in each study group were euthanized, and lungs and nasal turbinates were harvested to determine viral loads by plaque-reduction assay (Fig. 5a, b) and the number of viral copies by RT-qPCR assay (Fig. 5c, d). In the control group three days post infection, high virus load was detected in the lungs of all animals (Fig. 5a), ranging from 6.5 × 10^5^ PFU/g to 2.5 × 10^6^ PFU/g, and in the nasal turbinates of all but one animal ranging from 4.0 × 10^2^ PFU/g to 1.2 × 10^4^ PFU/g (Fig. 5b). In contrast, no virus was detected in the lungs of four out of five vaccinated hamsters, while the remaining one had the viral titer 645-fold less than in the control group. No virus was detected in the nasal turbinates of two vaccinated animals, while the three remaining animals displayed titers reduced by 57-fold compared with the control animals. On day 15, no SARS-CoV-2 was detected in lungs and nasal turbinates of both the control and the vaccinated hamsters (Fig. 5a, b).

RNA isolated from the lungs and nasal turbinate homogenates were assessed for the presence of viral RNA copies by RT-qPCR assay (Fig. 5c, d). In the control group, high-virus RNA copies were detected in the lungs and nasal turbinates of animals with ~ 1 × 10^11^ RNA copies/g on day three post challenge, ~ 1 × 10^7^ RNA copies/g in the lung and ~ 6 × 10^7^ RNA copies/g in the nasal turbinates on day 15 post challenge. In contrast, significantly lower viral RNA copies were detected in the ConVac-vaccinated animals with ~ 7 × 10^7^ RNA copies/g in the lungs and ~ 1 × 10^10^ RNA copies/g in the nasal turbinates on day three post challenge, ~ 3.6 × 10^6^ RNA copies/g in the lungs, and ~ 5 × 10^6^ RNA copies/g in the nasal turbinates on day 15 post challenge. We assume that a portion of the detected viral RNA on day three is residual input challenge virus as it was below the detection limit of the plaque assay. In addition, the detected viral RNA on day 15 post challenge is not derived from viable viral particles since no plaques were detected.

### Vaccinated animals do not demonstrate enhanced lung pathology upon challenge

**Fig. 6:**
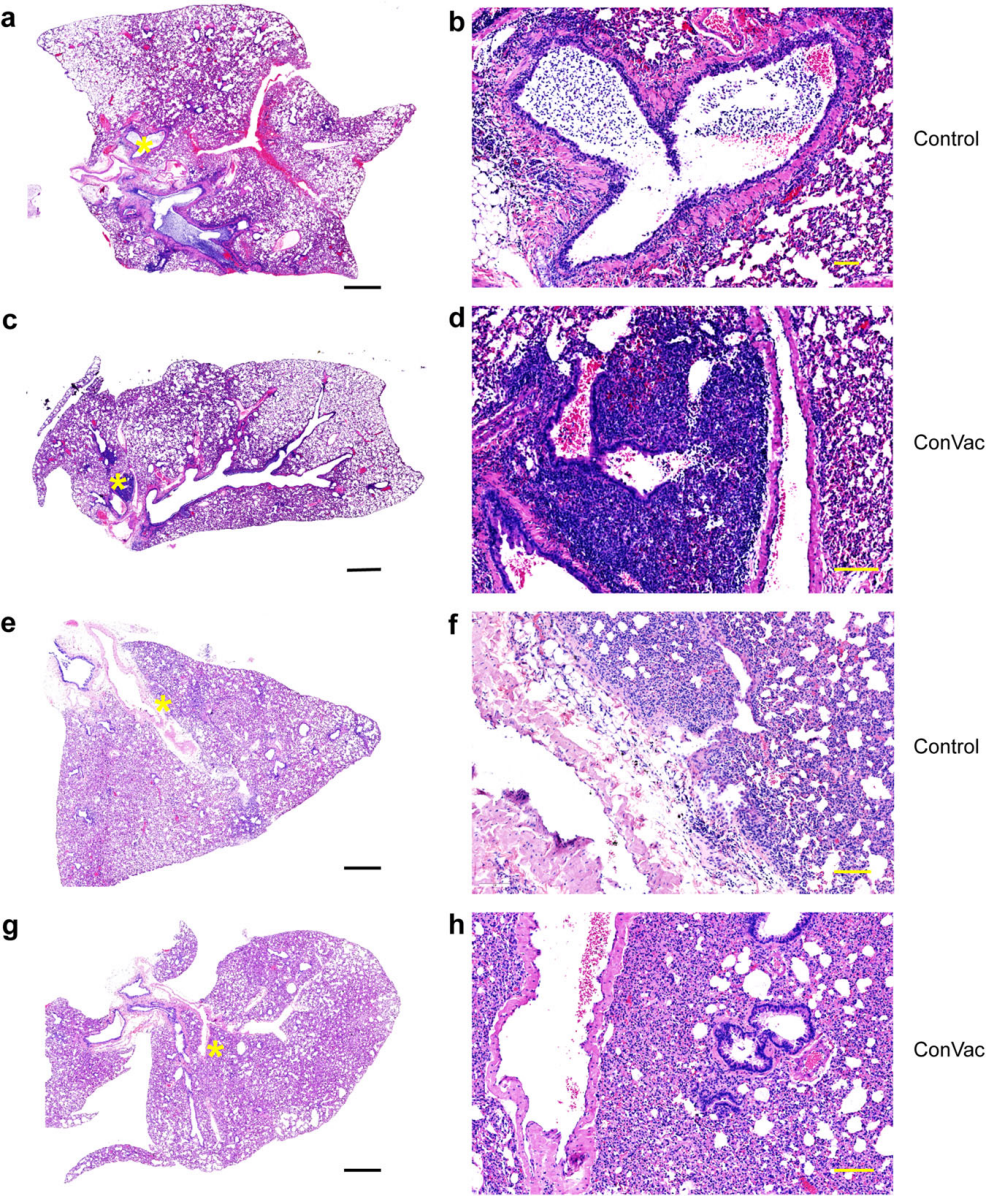
SARS-CoV2 lung pathology. Representative histological images of SARS-CoV-2 infection in control and vaccinated hamster lungs. Left panels: low magnification, right panels: higher magnification of selected regions, indicated by asterisks (yellow) on low-magnification images. **a**, **b** Day-3 control: widespread consolidation and inflammatory infiltration is clearly visible, along with obstruction of airways by inflammatory cells. Higher magnification illustrates airway infiltration by mononuclear cells, septal thickening and airway occlusion. **c**, **d** Day-3 ConVac vaccine: moderate inflammatory changes with mononuclear cellular infiltration, septal thickening, and mild airway pathology. Higher magnification illustrates focal infiltration by inflammatory cells. **e**, **f** Day-15 control: typical interstitial pneumonic changes, endothelial damage and septal thickening. Higher magnification illustrates endothelial damage and airway epithelia hyperplasia. **g**, **h** Day-15 ConVac vaccine: moderate interstitial pneumonia with septal thickening and endothelial damage. The magnified image illustrates airway cellular infiltration and endothelial damage. Scale bars are 1 mm in the left panels and 0.1 mm in the right panels.

**Fig. 7:**
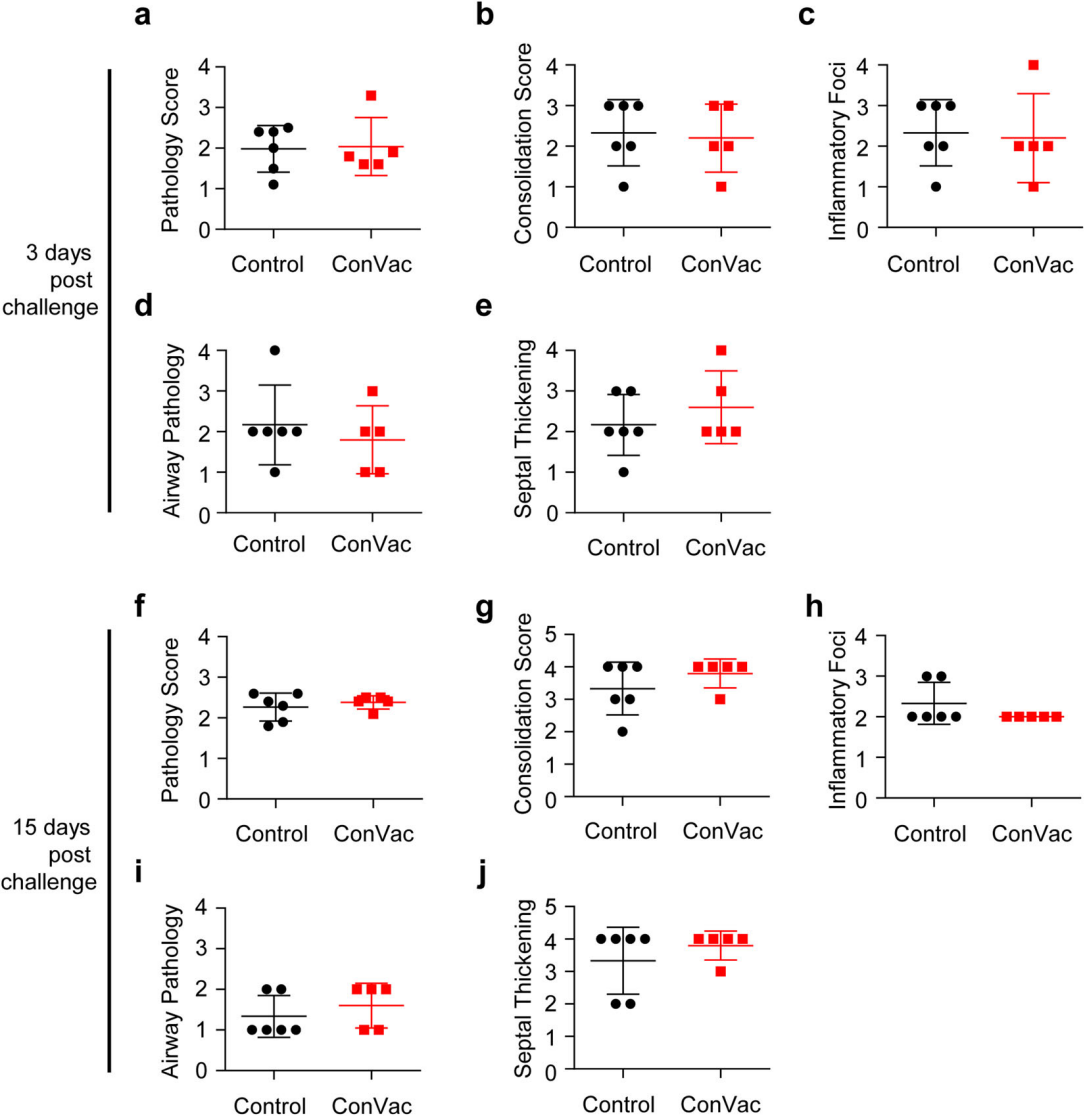
Comparative pathology scores for lungs from vaccinated and control hamsters post SARS-CoV-2 challenge. Scores at three days (**a**-**e**) and 15 days (**f**–**j**) post challenge. Scores for overall lung pathology (**a**, **f**), and individual criteria, including consolidation or extent of inflammation (**b**, **g**), type of inflammatory foci (**c**, **h**), airway pathology (**d**, **i**) and septal thickening (**e**, **j**), are displayed. The pathology scores (mean) were calculated based on the criteria described in Supplementary Table 1. The ConVac vaccine group is shown in red and the control group in black. Data represent mean ± SD, *N* = 6 for control group and *N* = 5 for ConVac vaccine group at each time point.

Pathology scores were determined in a blinded manner in lung sections of control and vaccinated animals on days three and 15 post challenge. Interstitial pneumonic changes and septal thickening were noted in control and vaccinated animals (Figs. 6 and 7). On day three, the control group (Fig. 6a, b) showed widespread inflammatory changes and obstruction of airways by inflammatory cells, while the ConVac vaccine group (Fig. 6c,d) had moderate inflammatory changes, septal thickening, and mild airway pathology. On day 15 (Fig. 6e, f), the control unvaccinated group displayed typical interstitial pneumonic changes and endothelial damage, while the ConVac vaccine group (Fig. 6g, h) displayed interstitial pneumonia with septal thickening and reduced mononuclear cellular infiltrates in airways. A semiquantitative comparison (Fig. 7) of the control and vaccinated animals demonstrated no enhanced pathology in the vaccinated group (*P* > 0.05 for all panels, Mann–Whitney test).

## Discussion

The VSV vaccine platform has several features that make it a strong candidate for further development. These include the ability to stably express an inserted foreign open-reading frame, the robust replication resulting in a high immunogenicity, and the lack of preexisting immunity in the human population. VSV-vectored vaccine candidates have been developed against several human pathogens^24^, including Ebola virus; in 2019, the VSV-vectored vaccine Ervebo was approved by the European Medicines Agency and the FDA. Here we designed and developed a VSV-based SARS-CoV-2 vaccine ConVac and tested it for immunogenicity and efficacy in the hamster model of COVID-19.

The ConVac vaccine elicited a robust humoral response and protected hamsters from SARS-CoV-2 replication in the lower respiratory tract. A single dose of ConVac elicited strong SARS-CoV-2 S1-binding and virus-neutralizing antibody titers. Importantly, the ConVac vaccine induced neutralizing antibodies against the B.1.1.7 virus (the UK variant), a result consistent with the data obtained with other vaccine candidates, including the mRNA vaccines BNT162b2 from Pfizer^23^ and mRNA-1273 from Moderna^25^. We note that two vaccinated animals were euthanized on days 10 and 11 after immunization (i.e., prior to the SARS-CoV-2 challenge) due to a disease of unknown etiology; however, we cannot completely exclude a residual neurovirulence of VSV G at the high, 2 × 10^7^ FFU, vaccine dose used in our study. SARS-CoV-2 viral loads determined by plaque assay in lung tissues were significantly reduced in ConVac-vaccinated animals compared with controls, with only one vaccinated hamster having detectable viral loads on day three, suggesting that the ConVac vaccine prevents or strongly reduces SARS-CoV-2 replication in the lower respiratory tract. The hamster with the detectable viral load on day three had the lowest neutralizing antibody titer. Viral loads in the nasal turbinate tissues of vaccinated hamsters were reduced compared with control animals, but the difference was not statistically significant due to the limited number of animals per group and high variability between samples. Viral RNA levels determined by RT-qPCR confirmed these findings of a more robust protection in the lung compared with the nasal turbinates. These data suggest that ConVac elicited an immune response to effectively control the viral load.

Two recent studies used G gene-deleted VSV vaccine constructs expressing full-length SARS-CoV-2 S protein in mice and hamsters^26,27^, the same approach as used by Merck, which recently discontinued the development of SARS-CoV-2 vaccine candidate V590 due to its low immunogenicity in humans^28^. In contrast to this strategy, we used only the S1 subunit of the protein because it contains the necessary neutralizing epitopes with that in the receptor-binding domain being highly protective^13^, and both prefusion and postfusion S2 conformations present potential drawbacks as vaccine constructs^14^. Furthermore, unlike the S1 protein, the S2 subunit is more conserved between coronaviruses possibly leading to predominant S2-specific responses in humans previously infected with heterologous coronaviruses. Indeed, while cross-neutralizing antibodies targeting S2 have been identified in SARS-CoV-2-positive and -negative patient sera^29–31^, these have not been shown yet to prevent or treat COVID-19 in contrast to S1-specific monoclonal treatments^32,33^. The 50% mean-neutralizing titer induced at day 28 by our vaccine, 1:369, was higher than the titer of 1:173 elicited by the other VSV vaccine candidate assessed in hamsters by Yahalom-Ronen et al.^27^. When tested in mice, one of these vaccines elicited mean- and median-neutralizing titers greater than 1:5000 after two vaccine doses but the median neutralization titer after one vaccine dose was only 1:59^26^. The difference in the immunogenicity can also be explained by differences in growth kinetics and tissue tropism between a G-deleted VSV and a G-containing virus with the latter growing faster, having a broader tropism in vivo and targeting additional types of immune cells. Side-by-side comparisons would be needed to elucidate this further.

We used the hamster model of COVID-19, which does not require SARS-CoV-2 adaptation, exhibiting high viral titers and severe pathologic changes in the lungs^10–12^, and therefore seems to reproduce a severe form of COVID-19 observed in humans. Indeed, we confirmed that SARS-CoV-2 intranasal infection of hamsters results in viral replication in the upper and lower respiratory tract. Our data are in agreement with Imai et al.^10^, who showed higher viral loads in lungs than in nasal turbinates whereas Chan et al. observed the opposite results^11^. Observed lung histopathological damage was consistent with the changes described by others as this paper was in preparation^10–12^. Therefore the golden Syrian hamster represents an excellent model of a severe COVID-19 suitable for vaccine testing.

Besides hamsters, several animal models have been developed for COVID-19. These include the mouse model with the mouse-adapted SARS-CoV-2^34,35^, transgenic mice expressing human angiotensin-converting enzyme 2 (ACE-2)^36^, mice infected with a replication-defective Ad5 adenovirus expressing ACE-2 (Ad5-hACE2) transiently^37^, and the NHP model of COVID-19^38^. The mouse-adapted strains of SARS-CoV-2, despite reaching the titers of 10^6^ PFU/g or 10^8.3^ viral RNA copies per gram of lung tissue, caused only mild-to-moderate lung disease^34,35^. In the Ad5-hACE2 transduced mice, SARS-CoV-2 replicated in the lungs reaching 10^6^ PFU/g and caused a lung pathology consistent with a severe pneumonia^37^. Transgenic mice expressing human ACE2 supported SARS-CoV-2 replication in the lungs (10^6.8^ copies/ml) and developed moderate interstitial pneumonia^36^. In Rhesus macaques, SARS-CoV-2 replicated in the lungs reaching 10^5^–10^8^ copies/g but induced only mild-to-moderate interstitial pneumonia^38^. Thus, the levels of pathology induced by SARS-CoV-2 in the available animal models vary significantly from very mild to severe with the hamster representing a model of a severe disease, potentially explaining some residual lung pathology observed in the ConVac-vaccinated hamsters. Indeed, we propose that despite the lack of detectable viable SARS-CoV-2 by plaque assay, the observed pathologic changes in ConVac-vaccinated hamsters are likely to be related to the extremely high susceptibility of hamsters to SARS-CoV-2 on one hand and the very robust immune response to the vaccine on the other. Upon intranasal challenge, binding to cells of the epithelium of the respiratory tract and possibly some very limited replication, the virus is likely to be neutralized by the high levels of antibodies, resulting in the influx of Fc-bearing immune cells including lymphocytes and macrophages. Importantly, while some pathologic changes were observed in vaccinated animals, no enhancement compared with the control group was detected. Furthermore, testing of a vaccine in different animal models (mouse, hamster, ferret, and NHP) with various levels of susceptibility to SARS-CoV-2 is likely to result in a more realistic prediction of the outcome of vaccination and subsequent infection in humans.

In conclusion, our data show that the VSV-vectored SARS-CoV-2 vaccine based only on the S1 subunit induces a high titer of serum-neutralizing antibodies and protects hamsters from SARS-CoV-2 replication in the lower respiratory tract without causing an enhanced disease.

## Methods

### Antibodies

Monoclonal antibody CR3022 was produced by transient transfection of 293 F cells with cDNA expression plasmids obtained from BEI resources. The cells were transfected at a density of approximately 2 × 10^6^ cells/ml with equal amounts of each plasmid using Expifectamine reagent (Thermo Fisher) according to the instructions provided by the manufacturer. The supernatant was harvested after 4–5 days and antibody purified on Protein G Sepharose (Abcam, GE Healthcare) following standard protocols. Monoclonal antibodies I1 and I14 against the VSV glycoprotein and monoclonal antibody 23H12 against the VSV matrix protein were purified from hybridoma supernatant by affinity chromatography on Protein G sepharose. The antibodies were labeled with fluorophores using Dylight antibody labeling kits from Thermo Fisher. The following SARS-CoV-2-specific human monoclonal antibodies were kindly provided by Distributed Bio: DB_A03-09, 12; DB_B01-04, B07-10, 12; DB_C01-05, 07, 09, 10; DB_D01, 02; DB_E01-04, 06, 07; DB_F02-03.

### Generation of ConVac vaccine

Codon-optimized cDNA encoding the SARS-CoV-2 S1 domain fused to the C-terminal portion of the VSV glycoprotein was obtained from Genscript. The cDNA was cloned into the XhoI and NheI sites of a modified recombinant VSV vector containing an additional transcription start–stop signal between the G and L genes^9^. The recombinant virus was recovered on 293 T cells as described previously, filtered through a 0.22-µm filter and used to inoculate Vero (CCL-81, ATCC) or human BEAS-2B lung cells (gift from R. Plemper, University of Georgia). The infected cells were cultured in serum-free VP-SFM or Optipro medium. Cell culture supernatant was harvested three days post inoculation, filtered through 0.45-um PES membrane filters and used for virus characterization and animal experiments.

### Characterization of the ConVac vaccine

Baby hamster kidney (BSR) cells were inoculated with ConVac or VSV control virus expressing GFP at an MOI of 0.2 PFU. The following day, cells were lysed in detergent buffer (1% TritonX-100, 0.4% sodium deoxycholate, 150 mM NaCl, 20 mM HEPES, pH 7.0, and 1 mM EDTA) supplemented with a protease inhibitor cocktail (Thermo Fisher). Protein concentration was determined by BCA assay (Thermo Fisher), and ten micrograms of total protein were resolved on 4–20% Tris-Glycine gels (Thermo Fisher). The proteins were transferred to nitrocellulose membranes and probed with polyclonal rabbit serum against SARS-CoV S1 domain (Thermofisher, Cat No. PA5-81798 and PA5-81795), monoclonal antibodies I1 (8G5F11) and I14 (IE9F) against the VSV glycoprotein, and monoclonal antibody 23H12 against VSV matrix protein provided by Douglas Lyles (Wake Forest University). After incubation with HRP-conjugated anti-rabbit or anti-mouse IgG (Jackson Immunoresearch), the membrane was incubated with WestDura substrate (Thermo Fisher), and bands were visualized on a Fluochem M imager (Biotechne). All blots shown in Fig. 1c were derived from the same experiment and processed in parallel.

For immunofluorescence staining, Vero E6 cells were seeded on coverslips and infected at three different MOI ranging from 0.01 to 0.1 PFU. After 10 h, the cells were fixed for 10 min in Cytofix/Cytoperm (Becton Dickinson) and permeabilized for 10 min in 0.5% Tween 20 in Dulbecco phosphate-buffered saline (DPBS). After washing with DPBS, the cells were incubated for 2 h with monoclonal antibody CR3022 conjugated to Dylight 550 or a mixture of monoclonal antibodies I1 and I14 conjugated to Dylight 488. Coverslips were washed in DPBS and mounted using Prolong Glass (Thermo Fisher). Images were acquired on a Zeiss Axioskop40 microscope equipped with a ProgresCF digital camera.

To assess viral replication of ConVac in vitro, Vero (CCL-81) cells were seeded in 6-well plates and infected the next day at 34 °C at an MOI of five and 0.05 PFU. After 1.5 h the inoculum was removed the cells were washed 3x in DMEM and fresh medium containing 2% FCS was added to each well. The supernatant was collected at various time points from 6 to 36 h post infection. A tenfold serial dilution of supernatant was prepared in DMEM medium in a 96-well plate. The diluted supernatant was added to Vero E6 cells seeded in 96-well plates the day before. After 24 h of incubation at 34 °C, the cells were fixed with 80% acetone and stained with fluorescently labeled monoclonal antibody 23H12 against the VSV matrix protein.

### Viruses

The SARS-CoV-2 challenge strain used in this study is the first U.S. isolate SARS-CoV-2 USA-WA1/2020 from the Washington State patient identified on January 22, 2020^20^. Passage 3 was obtained from the World Reference Center for Emerging Viruses and Arboviruses (WRCEVA) at UTMB. Virus stocks were propagated in Vero E6 cells. The challenge stock used in this study is passage 5. The recombinant SARS-CoV-2-expressing Neon Green protein (SARS-CoV-2-mNG)^22^ used in the neutralization assay was developed by Dr. Pei-Yong Shi at UTMB. Virus stocks were propagated in Vero E6 cells and passage 4 was used in this study. The recombinant USA-WA1/2020 SARS-CoV-2 viruses expressing the spike protein of the United Kingdom variant (B.1.1.7) used in neutralization assays were developed in our previous study^23^. Virus stocks were propagated in Vero E6 cells, and passage-1 stocks were used for neutralization assays.

### Animal studies

The studies were carried out in strict accordance with the recommendations described in the Guide for the Care and Use of Laboratory Animals of the National Research Council. UTMB is an AAALAC-accredited institution and all animal work was approved by the IACUC Committee of UTMB. All efforts were made to minimize animal suffering and all procedures involving potential pain were performed with the appropriate anesthetic or analgesic. The number of hamsters used was scientifically justified based on statistical analyses of virological and immunological outcomes.

### Pathogenicity of SARS-CoV-2 in hamsters

On day zero, seven-week-old golden Syrian female hamsters (Envigo) were anesthetized with ketamine/xylazine, and eight animals were exposed intranasally to the targeted dose of 10^5^ PFU of SARS-CoV-2 in a volume of 100 µl, while four animals were mock-infected with 100 µl of 1X DPBS. Animals were monitored daily for weight loss and signs of disease. Half of the animals in each group (four SARS-CoV-2-infected and two mock-infected hamsters) were euthanized by CO_2_ inhalation for viral load determination two and four days post challenge. Lungs collected on days two and four from two animals in each group were stored in 10% formalin for histopathological assessment (see below).

### Vaccination and SARS-CoV-2 challenge

Seven-week-old golden Syrian female hamsters (Envigo) were anesthetized with 5% isoflurane prior to immunization and blood collections and with ketamine/xylazine prior to the SARS-CoV-2 challenge. On day zero, the vaccine group (*N* = 12 animals) was inoculated with 2 × 10^7^ FFU of ConVac in 100 µl of injection volume via the intramuscular route (50 µl per hind leg), while the control group (*N* = 12 animals) remained naive. Vena cava blood collections were performed one day prior to the immunization and on day 28 afterward. On day 31, vaccinated and control animals were exposed intranasally to the targeted dose of 10^5^ PFU of isolate SARS-CoV-2. Animals were monitored daily for weight loss and signs of disease. Half of the animals in each group (five vaccinated and six control hamsters) were euthanized by overdose of injectable anesthetics three days post challenge for viral load determination. The remaining animals were euthanized 15 days post infection by overdose of ketamine/xylazine.

### Binding antibody response

To determine antibody responses to the S protein of SARS-CoV-2, an indirect ELISA was developed utilizing purified S1 protein. The soluble S1 protein was produced by transfecting 293 T cells with a plasmid that expresses a secreted S1 ectodomain (aa 16–681) fused to the C-terminal HA tag. Purification of the HA-tagged protein from the supernatant of transfected cells was carried out as described previously^19^. Antibody responses to SARS-CoV-2 spike protein (S1) were measured by an indirect ELISA as described previously^19^. Briefly, wells were coated overnight at 4 °C with 500 ng/mL of S1-recombinant protein. The secondary antibodies used in the ELISA are HRP-conjugated goat anti-syrian hamster IgG secondary antibody (Jackson immunoresearch, Cat# 107-035-142, 1:8000 in PBST) or mouse anti-hamster IgG2/3-HRP (Southern Biotech, Cat# 1935-05, 1:8000 in PBST). Optical density was measured at 490 nm and 630 nm using an ELX800 plate reader (Biotek Instruments). Data were analyzed with GraphPad Prism (Version 8.4.3) using 4-parameter nonlinear regression.

### Neutralizing antibody response

Sera collected from animals were tested for neutralizing capabilities against SARS-CoV-2. Neutralization assay medium is MEM (Gibco) supplemented with 2% FBS (Gibco), 50 µg/ml gentamicin sulfate (Corning) and 5 mM Hepes buffer (Corning). Briefly, serum samples were heat-inacitvated (30 min at 56 °C). Tenfold diluted sera were further diluted in a 2-fold serial fashion, and 60 µl of each serum dilution was mixed with 60 µl of SARS-CoV-2-mNG (200 PFU)^22^. The serum/virus mixtures were incubated for 1 h at 37 °C. About 100 µl of the serum/virus mixtures were then transferred to Vero E6 cell monolayers in flat-bottom 96-well plates and incubated for two days at 37 °C. Virus fluorescence was measured with a Cytation Hybrid Multi-Mode reader at 488 nm (Biotek Instruments). For the neutralization of the UK (B.1.1.7) virus^23^, nine out 10 serum samples from the ConVac group and 10 out 12 serum samples from the control group were available. Serum samples were heat-inactivated (30 min at 56 °C), diluted 10-fold and further diluted in a 2-fold serial fashion, and 50 µl of each serum dilution was mixed with 50 µl of virus added for the targeted number of 50 PFU. The serum/virus mixtures were incubated for 1 h at 37 °C. Fifty µl of the serum/virus mixtures were then transferred to Vero E6 cell monolayers in flat-bottom 96-well plates, incubated for 1 h at 37 °C, and the serum/virus mixture was replaced with 2:1 overlay composed of 1% methylcellulose (Fisher Chemical) and MEM (Gibco) supplemented with gentamicin sulfate (Corning) and 4% FBS (Gibco). Plates were incubated two days at 37 °C, fixed with 10% neutral buffered formalin (Fisherbrand), and removed from biocontainment. Plates were washed three times with DPBS (Corning). Plaques were visualized by immunostaining with a primary antibody cocktail of 37 SARS-CoV-2-specific human antibodies kindly provided by Distributed Bio. The primary antibody cocktail was diluted at 2 µg/ml in blotto (DPBS [Corning] with 5% milk), and plates were incubated for 1 h at 37 °C. Plates were washed three times in 1X DPBS (Corning). As secondary antibody, HRP-labeled goat anti-human IgG (SeraCare) was used at dilution 1:500 in blotto and plates were incubated for 1 h at 37 °C. Plates were washed three times in 1X DPBS (Corning) and plaques were revealed by a 30-min incubation at 37 °C with AEC substrate (enQuire Bioreagents).

### Tissue processing and viral load determination

For the pathogenicity study, animals from each study group were euthanized on days two and four post challenge, and the lungs and nasal turbinates were harvested. For the vaccine study, animals were euthanized on days three and 15 post challenge, and the lungs and nasal turbinates were harvested. For both studies, right lungs and nasal turbinates were placed in L15 medium supplemented with 10% fetal bovine serum (Gibco) and Antibiotic-Antimycotic solution (Gibco), flash-frozen in dry ice, and stored at −80 °C until processing. Tissues were thawed and homogenized using the TissueLyser II system (Qiagen). Tissue homogenates were titrated on Vero E6 cell monolayers in 24-well plates to determine viral loads. Plates were incubated three days at 37 °C before being fixed with 10% formalin and removed from containment. Plaques were visualized by immunostaining with 1 µg/mL cocktail of 37 SARS-CoV-2-specific human antibodies kindly provided by Distributed Bio. As the secondary antibody, HRP-labeled goat anti-human IgG (SeraCare) was used at dilution 1:500. Primary and secondary antibodies were diluted in 1X DPBS with 5% milk. Plaques were revealed by AEC substrate (enQuire Bioreagents).

### qRT-PCR

Tissue homogenates were mixed with TRIzol Reagent (Life Technologies) at a 1:5 volume ratio of homogenate to TRIzol. The RNA extraction protocol for biological fluids using TRIzol Reagent was followed until the phase-separation step. The remaining RNA extraction was done using the PureLink RNA Mini Kit (Ambion). The quantity and quality (260/280 ratios) of RNA extracted was measured using NanoDrop (Thermo Fisher). SARS-CoV-2 nucleoprotein cDNA was generated from RNA from Bei Resources (NR-52285) by One-Step RT PCR (SuperScript IV, Thermo Fisher) with primers SARS COV-2 N IVT F1 (5′-GAATTCTAATACGACTCACTATAGGGGATGTCTGATAATGGACCC-3′) and SARS COV-2 N IVT R1 (5′- GCTAGCTTAGGCCTGAGTTGAGTCAGCACTGCT-3′). The SARS-CoV-2 N standards were generated by in vitro transcription of the generated SARS-CoV-2 N cDNA using the MegaScript T7 Transcription kit (Invitrogen), followed by using the MEGAclear Transcription Clean-Up Kit. Aliquots of 2 × 10^10^ copies/µL were frozen at −80 °C. Five microliters of RNA per sample were run in triplicate, using the primers 2019-nCoV_N2-F (5′- TTACAAACATTGGCCGCAAA-3′), 2019-nCoV_N2-R (5′-GCGCG ACATTCCGAAGAA-3′) and probe 2019-nCoV_N2-P-FAM (5′-ACAATTTGCCCCCAGCGCTTCAG-3′).

### Histopathology

Following euthanasia, necropsy was performed, gross lesions were noted, and left lungs were harvested in 10% formalin for histopathological assessment. After a 24-h incubation at 4 °C, lungs were transferred to fresh 10% formalin for an additional 48-h incubation before removal from containment. Tissues were processed by standard histological procedures by the UTMB Anatomic Pathology Core, and 4-μm-thick sections were cut and stained with hematoxylin and eosin. Sections of lungs were examined for the extent of inflammation, type of inflammatory foci, and changes in alveoli/alveolar septa/airways/blood vessels in parallel with sections from uninfected or unvaccinated lungs. The blinded tissue sections were semiquantitatively scored for pathological lesions using the criteria described in Supplementary Table 1. Examination was performed with an Olympus CX43 microscope at magnification 40X for general observation and 100X magnification for detailed observation. Each section was scored independently by two trained lab members, and as scores were in agreement, only one set is presented.

### Statistical analyses

Statistical analyses were performed with GraphPad Prism for Windows (version 6.07). *P* < 0.05 was considered significant.

### Biocontainment work

Work with SARS-CoV-2 was performed in the BSL-3 and BSL-4 facilities of the Galveston National Laboratory according to approved standard operating protocols.

### Reporting summary

Further information on research design is available in the Nature Research Reporting Summary linked to this article.

## Supplementary information

Supplementary Information

Reporting Summary

## Data Availability

The datasets generated during the current study are available from the corresponding author on reasonable request.
