# Supplementary material for: A single dose of replication-competent VSV-vectored vaccine expressing SARS-CoV-2 S1 protects against virus replication in a hamster model of severe COVID-19
Source: NPJ Vaccines. 2021 Jul 22;6:91. doi: 10.1038/s41541-021-00352-1 (PMC8298481; doi:10.1038/s41541-021-00352-1)
Supplement: Supplementary file 1 — Supplementary Information [file 41541_2021_352_MOESM1_ESM.pdf]

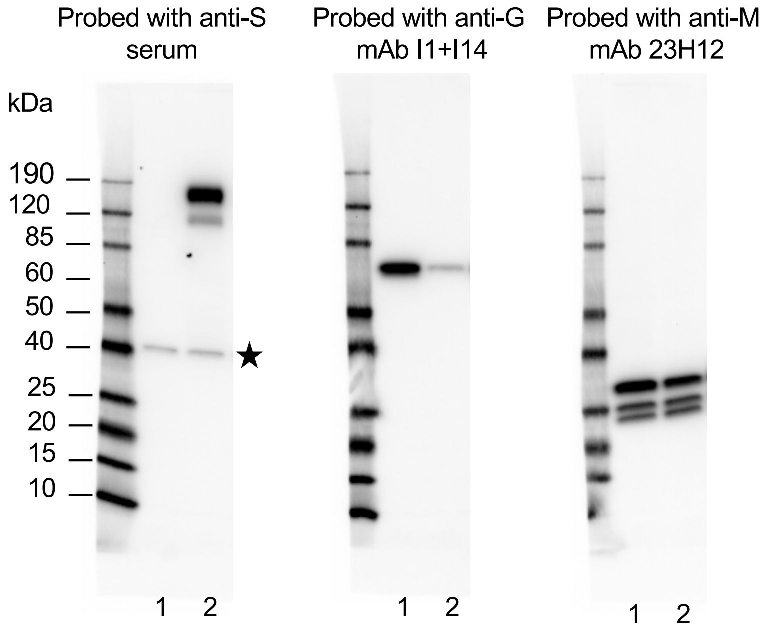

Supplemental Figure 1. Western blots. Western blot analysis of BSR cells infected with ConVac or a control VSV virus expressing GFP. Protein lysates were resolved on 4-20% polyacrylamide gradient gels and transferred to nitrocellulose membranes. Same samples and same amount were loaded in triplicate on same gel in the same order: Lane 1: VSV-GFP and Lane 2: Convac. The membranes were probed with polyclonal antiserum against the S1 domain (left panel), monoclonal antibodies against the VSV glycoprotein (middle panel) and a monoclonal antibody against the VSV matrix protein (right panel). The polyclonal antiserum directed against S1 detects an unspecific band of approximately 40 kDa which is identified by an asterisk (left panel).

Supplemental Table 1. Criteria for histopathology scoring

	**Scores→**	**0**	**1**	**2**	**3**	**4**
**A**	Extent of inflammation (% tissue involved)	0	<10	10-30	30-60	>60
**B**	Inflammatory foci type	No inflammation	Patchy inflammatory foci, few (<2)	Patchy inflammatory foci, many (>2)	Large inflammatory foci, few (<2)	Large inflammatory foci, many (>2)
**C**	Alveolar septa	Thin and delicate	Thickened in <10% HPF	Thickened in <30% HPF	Thickened in <60% HPF	Thickened in >60% HPF
**D**	Airways	Clear; no cells	Few cells in airway	Moderate cells in airway	More cells in air way; Epithelial hyperplasia	Occlusion of air way/epithelial hyperplasia or desquamation
**E**	Alveoli/ perivascular cuff/blood vessels/ pleuritis/cell types	Clear; no inflammatory cells	Few cells. Few PMN or MNC	Moderate cells/ PVC/mild congestion/ mild pleuritis/mostly MNC	More cells/PVC/ more congestion and pleuritis/more MNC and PMN	Abundant cells/large PVC/severe congestion or pleuritis/mixed cells

The criteria were adapted from Matute-Bello et al., 2011.

HPF – high power field (>10x); PMN – polymorphonuclear cells/heterophils; MNC – mononuclear cells including lymphocytes and macrophages; PVC – perivascular cuff.
