# Supplementary material for: A single dose of replication-competent VSV-vectored vaccine expressing SARS-CoV-2 S1 protects against virus replication in a hamster model of severe COVID-19
Source: NPJ Vaccines. 2021 Jul 22;6:91. doi: 10.1038/s41541-021-00352-1 (PMC8298481; doi:10.1038/s41541-021-00352-1)
Supplement: Supplementary file 2 — Reporting Summary [file 41541_2021_352_MOESM2_ESM.pdf]

nature research

Corresponding author(s): Alexander Bukreyev, PhD

Last updated by author(s): Apr 7, 2021

Reporting Summary

Nature Research wishes to improve the reproducibility of the work that we publish. This form provides structure for consistency and transparency in reporting. For further information on Nature Research policies, see our Editorial Policies and the Editorial Policy Checklist.

Statistics

For all statistical analyses, confirm that the following items are present in the figure legend, table legend, main text, or Methods section.

n/a	Confirmed	
☐	☒	The exact sample size (n) for each experimental group/condition, given as a discrete number and unit of measurement
☐	☒	A statement on whether measurements were taken from distinct samples or whether the same sample was measured repeatedly
☐	☒	The statistical test(s) used AND whether they are one- or two-sided<br>*Only common tests should be described solely by name; describe more complex techniques in the Methods section.*
☐	☒	A description of all covariates tested
☐	☒	A description of any assumptions or corrections, such as tests of normality and adjustment for multiple comparisons
☐	☒	A full description of the statistical parameters including central tendency (e.g. means) or other basic estimates (e.g. regression coefficient) AND variation (e.g. standard deviation) or associated estimates of uncertainty (e.g. confidence intervals)
☐	☒	For null hypothesis testing, the test statistic (e.g. F, t, r) with confidence intervals, effect sizes, degrees of freedom and P value noted<br>*Give P values as exact values whenever suitable.*
☒	☐	For Bayesian analysis, information on the choice of priors and Markov chain Monte Carlo settings
☒	☐	For hierarchical and complex designs, identification of the appropriate level for tests and full reporting of outcomes
☒	☐	Estimates of effect sizes (e.g. Cohen's d, Pearson's r), indicating how they were calculated

Our web collection on statistics for biologists contains articles on many of the points above.

Software and code

Policy information about availability of computer code

Data collection	N/A
Data analysis	Data analysis was performed with GraphPad Prism versions 6.07 and 8.4.3.

For manuscripts utilizing custom algorithms or software that are central to the research but not yet described in published literature, software must be made available to editors and reviewers. We strongly encourage code deposition in a community repository (e.g. GitHub). See the Nature Research guidelines for submitting code & software for further information.

Data

Policy information about availability of data

All manuscripts must include a data availability statement. This statement should provide the following information, where applicable:

- Accession codes, unique identifiers, or web links for publicly available datasets
- A list of figures that have associated raw data
- A description of any restrictions on data availability

The datasets generated during the current study are available from the corresponding author on reasonable request.

Field-specific reporting

Please select the one below that is the best fit for your research. If you are not sure, read the appropriate sections before making your selection.

☒ Life sciences ☐ Behavioural & social sciences ☐ Ecological, evolutionary & environmental sciences

For a reference copy of the document with all sections, see nature.com/documents/nr-reporting-summary-flat.pdf

Life sciences study design

All studies must disclose on these points even when the disclosure is negative.

Sample size	The number of samples was the minimum number required to obtain scientifically valid results
Data exclusions	No data were excluded from the analyses
Replication	All attempts at replication in the stated conditions were successful
Randomization	Allocation of animals to experimental groups was random
Blinding	Histopathology analysis was performed in a blinded manner otherwise there was no blinding

Reporting for specific materials, systems and methods

We require information from authors about some types of materials, experimental systems and methods used in many studies. Here, indicate whether each material, system or method listed is relevant to your study. If you are not sure if a list item applies to your research, read the appropriate section before selecting a response.

Materials & experimental systems

n/a	Involved in the study	
☐	☒	Antibodies
☐	☒	Eukaryotic cell lines
☒	☐	Palaeontology and archaeology
☐	☒	Animals and other organisms
☒	☐	Human research participants
☒	☐	Clinical data
☒	☐	Dual use research of concern

Methods

n/a	Involved in the study	
☒	☐	ChIP-seq
☒	☐	Flow cytometry
☒	☐	MRI-based neuroimaging

Antibodies

Antibodies used	Monoclonal antibodies CR3022, I1 and I14 produced in house as described. Monoclonal antibody 23H12 provided by Douglas Lyles (Wake Forest University). Anti-SARS-CoV-2 human monoclonal antibodies DB_A03-09, 12; DB_B01-04, B07-10, 12; DB_C01-05, 07, 09, 10; DB_D01, 02; DB_E01-04, 06, 07; DB_F02-03 provided by Distributed Bio. Polyclonal rabbit serum against SARS-CoV S1 domain (Thermofisher, Cat No. PA5-81798 and PA5-81795). HRP-conjugated anti-rabbit (Jackson Immunoresearch Cat No 711-035-152). Anti-mouse IgG (Jackson Immunoresearch Cat No 115-035-146), HRP-conjugated goat anti-syrian hamster IgG (Jackson immunoresearch, Cat No. 107-035-142), mouse anti-hamster-IgG2/3-HRP (Southern Biotech, Cat No. 1935-05). HRP-conjugated goat anti-human IgG (SeraCare Cat No. 474-1002).
Validation	See manufacturer's website for validation of commercial antibodies

Eukaryotic cell lines

Policy information about cell lines

Cell line source(s)	293F, 293T, Vero E6, Vero (CCL-81) and Baby hamster kidney (BSR) cell lines were obtained from the ATCC. Human BEAS-2B lung cells were provided by R. Plemper, University of Georgia.
Authentication	N/A
Mycoplasma contamination	Cell lines were tested for mycoplasma contamination with the MycoAlert Mycoplasma detection kit from Lonza
Commonly misidentified lines (See ICLAC register)	N/A

Animals and other organisms

Policy information about studies involving animals; ARRIVE guidelines recommended for reporting animal research

Laboratory animals	seven week-old golden Syrian female hamsters (Envigo)
Wild animals	N/A
Field-collected samples	N/A
Ethics oversight	Animal studies were reviewed and approved by UTMB IACUC.

Note that full information on the approval of the study protocol must also be provided in the manuscript.
